# Stress, plasticity, and fibrosis: unfolding the role of the IRE1**α**/RIDD/*Fgfr2* axis

**DOI:** 10.1172/JCI196740

**Published:** 2025-10-15

**Authors:** SeungHye Han

**Affiliations:** Division of Pulmonary and Critical Care, Department of Medicine, Feinberg School of Medicine, Northwestern University, Chicago, Illinois, USA.

## Abstract

Recent advances in sequencing technologies have enabled the identification of intermediate cell states during alveolar epithelial differentiation, which expand during repair following injury and in fibrotic lungs. Although ER stress has been implicated in pulmonary fibrosis, the underlying mechanisms remain elusive. The featured study by Auyeung and colleagues looked for links between the unfolded protein response sensor inositol-requiring enzyme 1α (IRE1α), intermediate epithelial cell states, and fibrotic remodeling in the lung. They identified Regulated IRE1-Dependent Decay (RIDD) as a key effector of IRE1α signaling that drives differentiation of alveolar epithelial type 2 cells to damage-associated intermediate cells and contributes to pulmonary fibrosis, likely by degrading *Fgfr2* mRNA. These findings unveil therapeutic targets and open new avenues for investigating the interplay between cellular stress responses, epithelial differentiation, and fibrotic disease.

Lung fibrosis is a progressive and complex process characterized by the replacement of the alveolar space with extracellular matrix, leading to tissue stiffening and, ultimately, to respiratory failure. Therapeutic options remain limited, and the few currently approved medications do not reverse disease progression or prolong survival. In this issue of the *JCI*, Auyeung et al. (1) examined the molecular drivers of intermediate epithelial cell states observed in injured and fibrotic lungs, focusing on a damage-associated transient progenitor (DATP) population associated with aberrant epithelial plasticity and lung fibrosis. Their observations reveal an important link between a downstream effector of ER stress and pathologic epithelial differentiation in the context of lung fibrosis.

## Intermediate transitional cells in the alveolar epithelium

Alveolar epithelial type 1 and 2 (AT1 and AT2) cells are the two main cell types comprising the lung alveolar epithelium, the primary site of gas exchange (2). AT1 cells are thin, membranous cells that cover most of the alveolar surface, enabling gas exchange by diffusion. In contrast, AT2 cells are cuboidal cells that synthesize and secrete surfactant to reduce alveolar surface tension. Numerous lineage-tracing studies have demonstrated that, in an in vivo context, AT2 cells function as stem cells with the capacity to self renew and differentiate into AT1 cells during homeostasis and upon injury.

During the transition of AT2 to AT1 cell type, an intermediate population of transitional epithelial cells appears. Although AT2 and AT1 cells typically exhibit mutually exclusive marker gene expression profiles, transitional cells coexpress both AT2 and AT1 marker genes and express genes associated with cell cycle checkpoints (e.g., *CDKN1A*) and cytokeratins (*Krt8* in mouse and *KRT17* in humans) (2). Under homeostatic conditions, intermediate transitional cells are rare in the alveolar epithelium, but they expand substantially during postnatal alveolar development, lung repair after injury, and in fibrotic lung disease. Transcriptomic profiling of these transitional cells showed enrichment of certain signaling and metabolic pathways, including MYC targets, oxidative phosphorylation, cellular stress response pathways, p53 pathway, and TGF-β signaling (3–5). Despite these insights, the exact molecular mechanisms governing transitional cell behavior and the factors that determine whether they support normal tissue repair or contribute to fibrotic remodeling remain poorly understood.

## Cellular stress response pathways and lung disease

**Figure 1:**
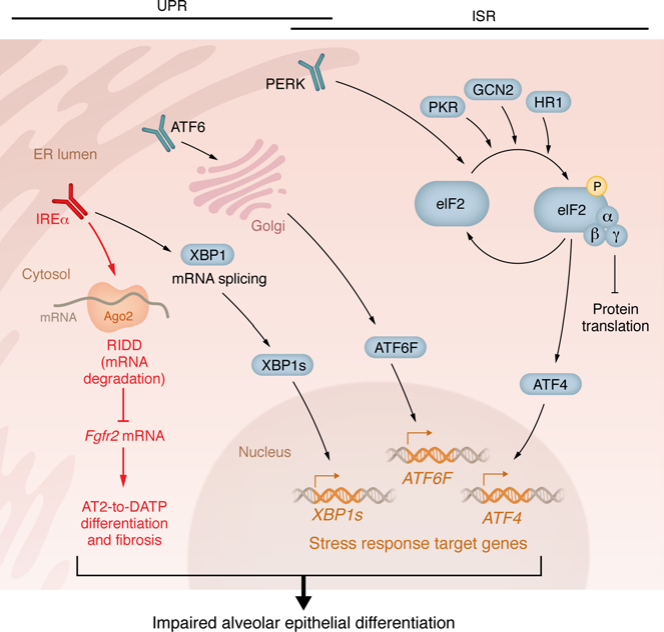
Cellular stress response pathways: the unfolded protein response and the integrated stress response. Cells respond to a variety of stressors by activating two major coordinated signaling networks to restore homeostasis: the unfolded protein response (UPR) and the integrated stress response (ISR). The UPR is activated when three ER sensors, ATF6, IRE1, and PERK, detect the accumulation of misfolded and unfolded proteins in the ER. Upon ER stress, ATF6 is transported to the Golgi apparatus, where it is cleaved. The released cytosolic fragment of ATF6 (ATF6f) then enters the nucleus and induces UPR gene expression. Upon activation, IRE1α dimerizes and autophosphorylates its kinase domain. The activated endoribonuclease domain excises a small intron from Xbp1 mRNA, producing XBP1s, a transcription factor that induces the UPR target genes. IRE1α also degrades select mRNAs (Regulated IRE1-dependent decay [RIDD]), ultimately reducing the protein-folding load. PERK activation leads to the phosphorylation of eukaryotic initiation factor 2 alpha (eIF2α), resulting in a global reduction in protein translation to prevent further ER burden, but paradoxically allowing selective translation of ATF4 that upregulates stress response genes. PERK is also a part of the ISR. The ISR integrates stress signals beyond the ER. In addition to PERK, three other kinases, PKR, HRI, and GCN2, can also phosphorylate eIF2α in response to viral infection, heme deficiency/mitochondrial dysfunction, and amino acid starvation, respectively. These inputs converge to modulate translation and reprogram gene expression, promoting cellular adaptation, or, if stress is severe, initiating programmed cell death. The study by Auyeung and colleagues (1) indicates that pathological cellular stress responses can impair alveolar epithelial differentiation.

Cellular stress response pathways are evolutionarily conserved signaling networks in eukaryotes that detect disruptions, such as protein misfolding, oxidative stress, nutrient deprivation, and infection, and orchestrate programs that restore homeostasis or eliminate irreparably damaged cells. The ER unfolded protein response (UPR) and the integrated stress response (ISR) are two central, interconnected signaling networks activated by these cellular disruptions (Figure 1).

The UPR activates when it senses accumulation of unfolded or misfolded proteins in the ER. Three sensors can initiate UPR: Inositol-requiring enzyme 1 alpha (IRE1α), protein kinase RNA-activated–like (PKR-like) ER kinase (PERK), and activating transcription factor 6 (ATF6) (6). UPR activation attenuates global protein translation to reduce ER-client protein load, upregulates molecular chaperones to enhance folding capacity, and enhances ER-associated degradation (ERAD) to clear misfolded polypeptides. Given the diversified outcomes of UPR activation, it follows that UPR sensors elicit crosstalk between multiple pathways. For instance, IRE1α activates the Xbp1s pathway, the JNK signaling cascade, and the regulated IRE1α-dependent decay (RIDD) pathway.

The ISR, on the other hand, is triggered by various cellular stressors, including viral infection, amino acid deprivation, mitochondrial dysfunction, and heme deficiency, as well as ER stress (7). Four kinases, PKR, GCN2, HRI, and PERK, function as stress sensors for the ISR; when activated, the ISR sensor phosphorylates eIF2α, reducing global protein translation yet paradoxically increasing translation of select transcription factors, such as ATF4, to induce stress response genes. If these stresses persist, apoptosis is initiated.

The UPR and ISR cellular stress response pathways have been implicated in the pathogenesis of pulmonary fibrosis, and targeting their activation has improved lung fibrosis in animal models (8–14). However, the precise molecular mechanisms underlying their effects on fibrosis remain unknown.

## Linking ER stress response-driven RIDD to transitional cells

In this issue of the JCI, Auyeung and colleagues (1) present elegant studies employing sophisticated genetic and pharmacological approaches to connect the ER stress sensor IRE1α and its downstream pathway, RIDD, to pathogenic AT2 cell plasticity that drives fibrotic remodeling in the lung. This group and others have previously demonstrated that IRE1α inhibitors alleviate lung fibrosis in animal models, and that mice with epithelial-specific IRE1α deletion are protected from bleomycin-induced fibrosis (10, 13, 14). Building on these findings, Auyeung and colleagues explored the downstream pathways of IRE1α in the pathogenesis of lung fibrosis, differentiating between the RIDD and Xbp1s pathways. Through analysis of single-cell RNA-seq data from multiple animal models, they revealed that IRE1α activation occurs only during fibrosis, not during postnatal alveolar development, in intermediate transitional cells, termed DATPs in this article. Using a small-molecule inhibitor that selectively inhibits the RIDD activity of IRE1α, they showed that RIDD, rather than Xbp1 splicing, is required for AT2-to-DATP differentiation in vitro and for the development of bleomycin-induced lung fibrosis in vivo in mice. They went on to show that fibroblast growth factor receptor 2 (*Fgfr2*) mRNA is a substrate of RIDD and provided in vitro evidence that the beneficial effect of the RIDD inhibitor in reducing AT2-to-DATP differentiation is mediated, at least in part, through preservation of *Fgfr2* transcripts.

## Impact and future directions

While prior studies have linked IRE1α-mediated UPR signaling to pulmonary fibrosis pathogenesis (10, 14) and recognized the potential role of FGFR2 in lung epithelial differentiation (15–17), this study is the first, to our knowledge, to identify that RIDD is the specific downstream effector of IRE1α-induced UPR that is critical to pathogenic AT2 cell plasticity and to suggest the link between *Fgfr2* and ER stress. This study advances the field through its finding that RIDD is the critical downstream effector driving IRE1α-induced AT2-to-DATP differentiation, likely by degrading *Fgfr2* mRNA. This finding not only deepens our understanding of UPR biology in lung disease but also highlights the potential of RIDD inhibition or strategies to preserve *Fgfr2* expression as promising, more specific therapeutic avenues. Future in vivo validation, including in *Fgfr2* overexpression models, will be crucial to translate these findings into targeted antifibrotic interventions.

It is noteworthy that the authors extended their findings beyond the alveolar epithelium to include the airway epithelium and even epithelial tissues in other organs such as the pancreas. Using in vitro culture systems, they demonstrated that the IRE1α/*Fgfr2* axis is conserved in both mouse tracheal basal cells and primary human pancreatic islet cells. These observations raise the possibility that stress response pathways, including the UPR, ISR, DNA damage response, p53 signaling, and TGF-β signaling, among others, may function as a shared regulatory network governing epithelial cell differentiation following injury. Crosstalk among these pathways could further refine and amplify their impact on epithelial plasticity. This concept, along with support from prior studies indicating that stress responses can influence epithelial plasticity (18–20), presents an exciting avenue for future investigation.

In conclusion, this study identifies a critical link between a IRE1α/RIDD/*Fgfr2* axis and pathogenic AT2 cell plasticity in pulmonary fibrosis. While further in vivo validation is needed to confirm the connection between UPR signaling and *Fgfr2*, these findings highlight promising opportunities for shaping therapeutic strategies.

## Version 1 10/15/2025

Electronic publication
